# Supplementary material for: Are carbon emissions trading and green financial instruments synergistic? -Comprehensive quantitative research based on content analysis
Source: PLoS One. 2024 Mar 7;19(3):e0298601. doi: 10.1371/journal.pone.0298601 (PMC10919601; doi:10.1371/journal.pone.0298601)
Supplement: S1 Data — (ZIP) [file pone.0298601.s001.zip › Data/地级市Z_F工作报告.zip!/╦─┤¿/╡┬╤⌠╩╨/└┤╫╘╣1⁄2╓┌║┼í╛┬φ┐╦╩2╛▌═°í┐_2018.docx]

各位代表：

现在，我代表市人民政府，向大会报告工作，请予审议，并请市政协委员提出意见。

2017年工作回顾

过去一年，面对复杂的经济形势和艰巨的改革发展任务，在省委、省政府和市委的坚强领导下，在市人大、市政协的监督支持下，我们坚持稳中求进工作总基调，以供给侧结构性改革为主线，开拓创新，奋发有为，扎实推进稳增长、促改革、调结构、惠民生、防风险各项工作，顺利完成市八届人大一次会议确定的目标任务，全市经济社会发展保持稳中奋进、进中向好的良好态势。

经济发展成效明显。地区生产总值1960.6亿元、增长9%，总量与增速均保持全省前列；一般公共预算收入106.2亿元、同口径增长10.2%。“项目年”工作扎实推进，全社会固定资产投资1311.4亿元、增长16.6%，130个省市重点项目全部完成年度投资目标，27个PPP项目吸引社会投资400亿元。规上工业增加值增长10.5%，完成技改投资470.7亿元、增长28.5%，医药增长19%,食品、机械增长均超过10%，战略性新兴产业产值789.1亿元、增长17.2%，30个数字化车间加快建设，入选国家工业运行重点联系城市、国家应急产业示范基地，“德阳通用航空创新型产业集群”成为国家创新型产业集群，20个特色园区全部挂牌。服务业增加值增长10%，社会消费品零售总额790.8亿元、增长13.2%，旅游收入285亿元、增长49.9%，中欧班列德阳现代物流港开通启运，共享经济、中高端消费、绿色低碳等新动能加快成长。金融机构各项存款余额2484.21亿元、增长6.73%，各项贷款余额1310.01亿元、增长9.29%，现代金融产业园入驻基金规模达420亿元。粮食实现“十四连增”，新建现代农业产业融合示范园区11个，旌阳原野牧场入选全国首批休闲观光牧场，广汉友谊村入选全国最美休闲渔村，中江被列为全国有机产品认证示范创建县。“三去一降一补”扎实推进，全市整体退出煤炭行业，淘汰落后产能38万吨，商品住房去化周期处于6个月历史低位，新三板挂牌企业13户、融资近8700万元，牵头建设天府（四川）股权交易中心“一带一路板”，我市企业在交易中心挂牌达616户。

改革创新纵深推进。全面创新改革取得重大成果，专利质押融资模式被国办发文推广，3项改革经验在全省推广。高新技术产业产值达1181.8亿元，科技对经济增长贡献率达55%。研制生产C919大飞机上百项关键件，完成50MW重型燃气轮机关键技术研发，成功研制“华龙一号”核能发电机。协同推动成德绵军民融合示范区建设，罗江金山镇入选全国军民融合特色小镇。新增省级企业技术中心9家，新增专利授权2727件、增长11%。引进创新团队13个、高端人才100余名，创造产值500多亿元。“装备制造工业云”入驻企业3500余家。成立科技企业孵化服务联盟，新增省级小企业创业示范基地2户，举办“德阳创客”活动20余场，新登记市场主体2.48万户、增长20%。全面深化改革280项规划任务完成80%，59项中央、省级重大试点全面展开。国资国企改革稳步推进，“僵尸企业”处置提速，“三供一业”分离移交加快推进。“放管服”改革持续深化，成为全省唯一全域启动相对集中行政许可权改革试点市，县级行政审批局全部挂牌运行，综合行政执法体制改革稳步推进，什邡被列为全国基层政务公开标准化规范化试点市。国家工商总局商标局德阳商标受理窗口正式运行，在全省率先实现“二十七证合一”。完成农村承包地确权颁证，广汉被确定为全国农村集体产权制度改革试点县，成都农交所德阳所挂牌营运。

城乡建设提质加速。完成城镇体系和城市总体规划，城乡空间规划体系日趋完善。成德同城化取得重大进展，签订《推动成德一体化发展合作备忘录》等一揽子协议，成德动车加密至半小时一列，实现西成客专经停德阳。新一轮交通大会战开局良好，天府大道北延线、德罗干道及北延线等项目加快建设，成都经济区环线高速德简段等项目全面开工，成德绵高速公路扩容等项目前期工作进展顺利，新（改）建农村公路710公里。“县县飞”直升机机场建设稳步推进。提档升级加快实施，“一谷八江十湖”生态田园城市概念规划研究基本完成，锦绣天府国际健康谷建设有序推进，实施城市提档升级项目17个，庐山南路、天山南路等干道改造完成，凯江桥西侧下穿等项目加快推进，新建环保公厕10座，成为全国首批生活垃圾分类示范城市。城市品质不断提升，顺利通过国家卫生城市复审，积极引进北京外国语大学、四川万汇等教育项目，建成全省首条移动支付示范街，政务云、智慧医疗、智慧教育、数字城管等进一步完善。全域城镇化取得重大进展，罗江区正式挂牌，双百大城市格局基本成形，启动广汉改区筹备工作，全市城镇化率达51%，新增建成区面积4平方公里。绵竹成为国家全域旅游示范区，白马关入选全国首批运动休闲特色小镇，创建省级特色小镇3个、省级“四好村”100个。

开放合作力度空前。成功举办建市以来规格最高、规模最大展览会——首届四川国际航空航天展览会。荣获《魅力中国城》优秀魅力城市，城市知名度、影响力大幅提升。举办“三星堆&丝绸之路”中国文化遗产峰会、第四届中国职业教育创新发展大会暨“一带一路”与职业教育国际峰会等大型活动。高分通过国家级出口高端装备智能制造产品质量安全示范区创建，鱼油等新产品助推外贸转型，完成进出口总额104亿元，综保区申报加快推进。积极参加西部投资大会、中外知名企业四川行、厦洽会等投促活动，新签约5亿元以上重大项目55个，到位市外资金722.1亿元。总投资81亿元的中德资源循环利用产业园项目成功签约。德国复材谷产业联盟中国分支机构——中国复材谷产业联盟在我市成立。全省“一带一路”国际合作项目代表——《吴哥王朝》在柬埔寨投入运营。成立“大九寨环线”旅游黄金走廊区域联盟，成功举办“两汉三国·秦蜀古道”定向运动城市邀请赛。外事、侨务、对台工作取得积极进展，海峡两岸产业合作区德阳产业园正式挂牌。

生态环境明显改善。圆满完成中央环保督察迎检工作，办结交办案件435件、办结率100%，封停矿井236个，关停“散乱污”企业1540家，完成磷石膏堆场整治14个。实施污染防治“三大战役”项目109个，全部淘汰中心城区燃煤锅炉，淘汰“黄标车”和老旧汽车6580辆，新增新能源公交车128辆，空气优良天数达247天、增加5天，两年来首次实现月度优良率100%。全面落实“河长制”，沱江、涪江流域德阳段水环境有效改善。72个城镇污水处理厂（站）开工建设，穿城堰、丁家堰等黑臭水体整治有效推进，全面消除劣V类水质，市县两级饮用水水质达标率100%。建成重金属土壤污染治理示范基地50亩。环保警察实现全域覆盖。农村垃圾生态处理“罗江模式”全国推广。新增森林面积0.81万亩，森林覆盖率24.45%。国家节能减排示范城市连续两年评为优秀，44个典型示范项目拉动投资76亿元，经开区国家循环化改造示范试点园区加快建设，在全省率先试行绿色建筑全周期闭合管理。

民生福祉持续增进。十大民生工程和20件民生实事全面完成，城乡居民人均可支配收入分别增长8.4%、9%。脱贫攻坚成效显著，157个市级贫困村全部退出，减贫1.6万人，贫困发生率降至0.77%。援藏援彝工作扎实开展，148个援建项目顺利推进，建成德阿骨关节病诊治中心。城镇新增就业4.47万人，城镇登记失业率3.91%。2014级民族地区“9+3”学生初次就业率达 99.49%。全民参保深入推进，各类社会保险参保率超过91%，城乡居民医疗保险政策范围内住院费报销比例超过75%，低保、优抚、退休人员基本养老金等标准进一步提高。新增（改造）养老床位3661张，建设日间照料中心100个。完成棚户区改造8549户，货币化安置率达95%。

社会事业全面进步。全域通过国家义务教育基本均衡发展督导评估认定，学前三年教育入园率达94%，新建幼儿园14所、小学1所，改善农村义务教育薄弱学校基本办学条件26所。四川城市轨道交通职业学院、西南财大天府学院罗江校区基本建成。医药卫生体制改革不断深化，“健康德阳”建设加快，国家医养结合试点稳步推进，公立医疗机构药品加成全面取消，分级诊疗全面实施，县域内就诊率达92.62%。公共文化服务体系不断健全，第三届三星堆戏剧季成功举办，2017年全省全民阅读活动在德阳启动。全民健身运动深入开展，市第三届体育运动会隆重举办，人均体育场地面积达1.78平方米，农村全民健身工程覆盖率达82%。“主动警务战略”深入实施。探索推行安全生产“两单一秘”制度，道路、生产、食品、药品等安全形势稳定。“德善之城”建设扎实推进。防灾减灾和应急处置能力巩固提升。国防动员、民兵预备役和“双拥”工作得到加强。

与此同时，我们积极开展“两学一做”学习教育和党的十九大精神学习宣传贯彻活动，进一步转变政府职能和作风。自觉接受市人大及其常委会法律监督、工作监督和市政协的民主监督，办理人大代表建议190件、政协提案322件，满意率达100%。政府立法工作取得突破，提请审议城市管理、物业管理两部地方性法规草案。全面启动“七五”普法，扎实推进“法律七进”。“互联网+政务服务”深入推进，实行智能化办公，行政效率大幅提升。持续保持正风反腐高压态势，“微腐败”整治成效明显，查处违纪违法案件979件。

统计调查、审计、档案、民族宗教、住房公积金、公共资源交易、妇女儿童、青年、老龄、残疾人、人防、保密、地方志、气象、红十字会等工作取得新进展。

各位代表！成绩来之不易，倾注着全市广大干部群众的心血和汗水，饱含着省委、省政府的关心和支持，更凝聚着社会各界的智慧和力量。在此，我代表市人民政府，向市人大代表、政协委员和离退休老同志，向各民主党派、工商联、无党派人士、人民团体和全市人民，向驻地人民解放军、武警官兵和公安干警，向一年来在各条战线辛勤工作、为德阳发展进步贡献力量的每一个人，表示衷心的感谢并致以崇高的敬意！

我们也清醒地看到，全市发展仍然面临不少困难和问题：发展不平衡不充分问题较为突出，发展质量和效益有待提升。重大产业项目引进少、落地难，投资营商环境有待改善。服务业“短板”明显，新兴产业支撑力不足，出口仍在下滑，创新能力有待提升。城市对人口特别是人才的吸引力、聚集力还不够强，城市功能品质仍需提高。资源环境约束日益趋紧，污染防治任务艰巨。财政持续增收面临诸多困难。市民文明素质有待提高。医疗、教育、养老等与群众期待还有差距。个别部门和少数干部仍存在贪安不为、拖沓推诿等现象。我们将正视这些问题，下大力气加以解决。

2018年工作安排

今年是贯彻党的十九大精神的开局之年，是改革开放40周年，是决胜全面建成小康社会、实施“十三五”规划承上启下的关键一年。当前，新一轮科技革命和产业变革正在蓬勃兴起，区域竞争和分工格局正在发生深刻变化。前景光明，挑战严峻。我们必须居安思危、站高谋远，勇于变革、勇于创新，以新面貌进入新时代、踏上新征程、干出新业绩，奋力开创新时代美丽繁荣和谐德阳新局面！

今年政府工作的总体要求是：坚定以习近平新时代中国特色社会主义思想为指导，全面贯彻党的十九大精神，全面落实省第十一次党代会、省委十一届二次全会和市第八次党代会、市委八届六次全会精神，始终保持专注发展、转型发展战略定力，坚持稳中求进工作总基调，坚持新发展理念，按照高质量发展要求，统筹推进“五位一体”总体布局和协调推进“四个全面”战略布局，坚持以供给侧结构性改革为主线，统筹做好稳增长、促改革、调结构、惠民生、防风险各项工作，加快建设“三高三优”经济强市，全力打好“三大攻坚战”，倾力打造“四张名片”，全面实施“十大重点工程”，促进全市经济社会持续健康发展，为决胜全面建成小康社会奠定坚实基础。

今年经济社会发展主要预期目标是：地区生产总值增长8.5%以上，总量突破2100亿元；全社会固定资产投资同口径增长15.5%以上；一般公共预算收入同口径增长9%；规上工业增加值增长9.5%；服务业增加值增长11%；社会消费品零售总额增长12%以上；城乡居民人均可支配收入分别增长8.5%以上、9%以上；节能减排达到国家和省的要求。

为实现上述目标，要重点做好以下工作：

一、加快建设世界智造之都，迈向经济高质量发展新阶段

坚定不移把发展作为第一要务，突出质量第一、效益优先，建设现代化经济体系，推动经济质量变革、效率变革、动力变革。

大力打造世界级先进制造业集群。深入实施“中国制造2025”，积极发展先进制造业，加快建设装备技术智能制造示范市。重点打造清洁能源、油气装备、航空专用装备等一批世界级先进制造业集群，实现规上装备制造工业总产值1800亿元。大力支持传统产业优化升级，实施设备换芯、机器换工、生产改线，打造装备制造、食品饮料、新型化工、生物医药“四大千亿产业集群”，加快推进211个2000万元以上的工业重点项目，传统产业主营业务收入超过3400亿元、增长15%。壮大战略性新兴产业，推动互联网、大数据、人工智能等数字经济与实体经济深度融合，推进铝空金属燃料电池、航空航天大型复杂结构件智能数字化车间、透平压缩机生产线等一批重大项目建设，优化通用航空、新能源、新材料等专业园区配套和服务环境，战略性新兴产业增加值增长18%以上。实施质量强市战略，积极创建全国质量强市示范城市，建设全国知名品牌示范区。

大力发展现代服务业。全面落实“1+1+N”服务业工作机制，着力构建现代服务业产业体系，优先发展电子商务、现代物流、科技服务、现代金融、文旅康养“五大新兴先导型服务业”。深入实施服务业“三三工程”，加大限上服务业企业培育力度，推动高端职业教育、新兴文化旅游、工业智能和设计服务创新突破。加快建设中欧班列德阳现代物流港、现代金融产业园、工农村特色商业街等服务业集聚区，抓好德阳世纪旌城教育医疗健康产业城、宝湾物流等项目落地。扩大健康养老、生态旅游、教育培训、文化体育等消费供给，创建“放心舒心消费城市”。聚焦服务实体经济、防控金融风险和深化金融改革三项任务，培育和支持100家企业挂牌上市，争创国家金融改革试验区，深化与地方法人金融机构、支付宝、微信等在移动支付领域合作，着力引进广发银行，金融业增加值突破100亿元。

大力实施乡村振兴战略。编制乡村振兴战略规划，实施乡村振兴“十大行动”。深化农业供给侧结构性改革，落实承包地“三权”分置改革，开展宅基地“三权”分置改革试点，推进农村集体产权制度改革，全面建成农村产权流转交易市场体系。建立农业信贷担保体系，落实支农政策。加快发展“三优两高”现代农业，抓好国家级现代农业产业园、农业综合体和农村产融发展示范园创建。稳定主要农产品供给，落实粮食安全行政首长责任制，粮食总产195万吨以上。提质升级乡村旅游，发展现代度假农庄、创意观光牧场、高端精品民宿等新业态，争取“田园综合体”省级试点，打造一二三产业融合发展示范园区10个，拓宽农民增收渠道。按照产业兴旺、生态宜居、乡风文明、治理有效、生活富裕的总要求，抓好“四好幸福美丽新村”示范带建设，推进“四好村”创建活动。完善农村基础设施，新（改）建农村公路400公里。加强懂农业、爱农村、爱农民“三农”工作队伍建设，探索建立新型职业农民制度。

二、加快建设国家创新型城市，再创德阳改革发展新优势

坚定不移把创新作为引领发展第一动力，坚决破除一切顽瘴痼疾，激活新动能、厚植新优势、打造新引擎。

系统推进全面创新改革。深入落实“9张清单”，继续总结提炼典型经验。开展军民融合攻坚，加强与12家军工集团和中物院的合作，建设特色军民融合产业园区，抓好航空产业配套基地、制造加工技术应用推广中心、军民融合工程创新中心等项目，积极创建成德绵国家军民融合创新发展示范区。开展创新发展攻坚，加快装备技术智能制造核心技术攻关，推动高端装备、通用航空、新能源汽车、新一代信息技术、机器人等突破发展，建设高端装备产业创新发展示范基地，高新技术产业产值突破1300亿元。开展人才创新攻坚，实施领军人才集聚计划，完善“1+3+N”政策体系，着力培养引进一批高素质创新人才和创新团队。深化专利质押融资模式，推广“银行贷款+保险保证+财政风险补偿”机制，促进科技、金融、产业“三融合”。推进专利、商标、版权“知识产权三合一”综合管理改革试点，力争专利申请量超过7500件。

打造特色鲜明的创新型城市。深入实施创新驱动发展战略，以科技创新带动产业创新、引领经济转型。强化顶层设计，瞄准世界科技前沿，完善科技创新规划，积极申报创建国家创新型城市，力争2020年进入创新型城市行列。提升科技创新能力，全社会研发投入占GDP比重达2.8％以上，新增省级以上重点实验室、企业技术中心、工程技术研究中心1-2个，建设西门子德阳高端装备智能制造创新中心、科技创新和知识产权服务中心等创新平台。培育壮大创新型企业集群，新增高新技术企业10家、科技型企业200家。继续办好“德阳创客”品牌活动，开展孵化载体提质增效行动。充分发挥各类市场主体活力，振兴实体经济，壮大民营经济，激发和保护企业家精神，弘扬工匠精神、劳模精神，鼓励更多社会主体投身创新创业。

扎实推进重点领域改革。深化“放管服”改革，持续推进相对集中行政许可权改革，扎实做好综合行政执法体制改革工作。深化商事制度改革，抓好“先照后证”改革，争取“证照分离”改革试点。加强事中事后监管，推动“双随机、一公开”全覆盖，深化行政执法“三项制度”试点，对新产业、新业态、新模式探索包容审慎的监管方式。做强做大投融资平台，探索建立全市一体化投融资体系，增强融资能力。大力推广政府和社会资本合作、特许经营、政府购买服务等方式，激发民间投资活力。巩固去产能成果，妥善处置“僵尸企业”，推进国有停产企业“人资分离”改革和“三供一业”分离移交工作。着力降低实体经济成本，实行收费目录清单管理，降低中介评估费用，扩大直购电直供气试点范围。加快推动德阳农商行挂牌运营，继续推进绵竹、什邡等县级农村信用联社改革。

三、加快建设成都北部新城，推动全域城镇化再上新台阶

大力实施区域协调发展战略，以成德同城化和全域城镇化的双发力、双提升，抢占西部大开发新格局的战略要地。

全力打好交通大会战。深入落实“项目年”部署，扎实推进35个省重点项目和233个市重点项目，重点推进“5高5快5轨”建设，构建“五环多轴”现代综合交通体系。强力推进天府大道北延线、成都经济区环线高速德阳段、什德中、德罗、德绵干道建设。力争启动成德绵高速公路扩容、连接各区市县的三环路、中心城区至各卫星城市第二干道、德阳至天府国际机场快速通道、成德间主要干道提档升级等项目。研究市域轨道交通示范线、成彭什绵轨道交通，全面对接成德轨道交通规划。

聚力深化成德同城发展。深入实施“六个协同”，加快实现“成德两地、如居一城”。积极对接成都城市总体规划，完成《成德一体化空间发展规划》，形成立体完整规划体系。规划建设城际列车南站，优化成德动车公交化运营。加快建设成德工业园、中国大车都成德配套产业园、成德电子商务产业合作园，强化中欧班列物流合作。深化旅游资源合作开发，共建区域旅游联盟，筹备第五届环龙门山骑游活动。全方位强化金融、卫生、教育、文化、社会保障等合作，共建优质都市生活圈。

全面提升城市功能品质。盘活释放土地资源，拓展城市发展空间。加强城市设计，争创省级“生态修复和城市修补试点城市”。大力实施提档升级，强化统筹施工，改造升级旌湖两岸、石刻公园、文庙广场、钟鼓楼广场，完成沱江路等17条干道提档升级，启动天府北湖、天府西湖、旌南湖、柳梢堰湿地公园等的建设，推进地下综合管廊、海绵城市建设，提升卫星城市特色魅力。着力提高现有教育医疗品质，大力引进名学校、名医院，推动成都实验外国语学校、北京外国语大学、四川万汇、四川天立等教育项目落地，促进华西医院、省人民医院、成都中医药大学、华西牙科集团等优质医疗资源向德阳延伸。搭建新型智慧城市“123N5”架构。抓好社区综合体、农贸市场、公交站场等公共配套设施建设。依法加强城市管理，提升城市环境质量，营造通透、整洁的城市空间。

深入推进全域新型城镇化。牢固树立“大德阳”理念，加快构建“四位一体”全域城镇体系。全面启动规划大会战，加快编制交通等11个专项规划，完成控规全覆盖，研究旌阳、罗江、广汉三个区域的一体化总体规划。按照全域市辖区格局，以广汉改区贯通成德绵都市区，有序推进什邡、绵竹改区。对标先进，竞位赶超，加快发展各具特色的县域经济。按照“十个一”标准提升小城镇承载力，力争创建全国特色小镇1个、省级特色小镇2个、百镇试点镇4个以上。促进房地产市场健康发展，构建多主体供给、多渠道保障、租购并举的住房制度，加快农业转移人口市民化，城镇化率达到52.5%。力争完成八角水库配套设施建设，加快华强沟、石泉水库建设，筹划沱江流域上游骨干水源工程建设。

四、加快建设国际文化名城，构建全面开放合作新格局

城市因文化而精彩。坚持以文化添魅力、以开放扩张力，激发文化创新创造活力，让德阳因灿烂的文化而享誉世界。

大力促进文化繁荣兴盛。全面践行社会主义核心价值观，深化“三讲三爱两进步”活动，着力提升市民文明素质，积极创建全国文明城市。深度挖掘三星堆文化、三国文化等资源，推动三星堆遗址、金牛古道申遗。突出打造《吴哥王朝》、德国院子和德阳院子等项目，加快建设三星堆文化、绵竹年画等产业园，创建国、省级文化产业示范园。大力发展文化创意产业，深度开发以三星堆等为题材的3D动漫、影视、网游，打造走向世界的文化产品。弘扬优秀传统文化，繁荣文艺创作，打造精品力作，培育创作人才。完善公共文化服务体系，实施文化惠民工程，高起点设计、高标准建设“四馆两中心”，举办好德阳音乐季、三星堆戏剧季、书香德阳全民阅读、绵竹年画节、罗江诗歌节等系列文化活动。

大力提升城市国际化水平。统筹利用国际国内两种资源，着力提升双向开放水平。推进产能国际化，发展国际产业合作园区，打响“清洁技术与新能源装备制造业国际示范城市”金字招牌，打造“一带一路”重要节点城市。推进教育国际化，办好中德汽车学院、库卡机器人学院。加快建设综保区，优化海关商检服务，用好出口企业综合金融服务平台，积极参加“万企出国门”“中国国际进口博览会”“西博会”等活动，促进外贸转型升级，实现进出口总额115亿元。大力改善投资营商环境，加快构建亲清新型政商关系，着力打通项目落地“中梗阻”“最后一公里”，力争到位市外资金740亿元。推动与波兰科宁市缔结友好城市。加快德阿产业园等合作园区建设，强化与长三角、珠三角和港澳台等的合作。

积极创建全域旅游示范区。大力推进“旅游+”融合发展，构建“全域全时全景全产业”旅游格局，实现旅游收入356亿元。全力打造锦绣天府国际健康谷，启动“三湾三谷、双创双东”起步区建设，大力推进五大湖区锦绣天府公园、凤翥路等一批重点项目。培育和引进知名文化旅游企业，推进三星堆文旅提升工程，开发凯江大回湾、中江石林谷等项目，打造四川绵竹国家玫瑰公园、白马关运动休闲小镇、雪茄风情小镇、高槐村田园综合体、西外乡美食公园等一批特色乡村旅游品牌。承办好第八届中国月季展、第六届四川温泉旅游节等特色活动。抓好旅游集散中心、网络平台、旅游交通、旅游厕所等配套设施建设。

五、加快建设生态田园典范，打造宜居宜业美丽新德阳

牢固树立和践行“绿水青山就是金山银山”理念，像对待生命一样对待生态环境，还自然以宁静、和谐、美丽。

打好污染防治攻坚战。狠抓中央、省环保督察反馈问题和自查自纠发现问题的全面整改，当好蓝天、绿水、净土、青山守护者。狠抓大气污染联防联控，推进高污染燃料锅炉淘汰和清洁能源改造，规范和整治“散乱污”企业，强化扬尘治理，加强重污染天气应急处置。深入落实“河长制”，大力开展黑臭水体整治，加强饮用水水源地保护，保障沱江涪江流域6大河流水质达标，促进饮用水更安全、旌湖水更清澈。狠抓土壤污染防治风险管控和修复试点，完成农业用地土壤污染源详查。开展第二次全国污染源普查。狠抓九顶山自然保护区、大熊猫国家公园拟建区域等重点区域环境治理，加强磷石膏污染整治和综合利用。强化环保执法能力建设，严守生态保护红线。

加快环保基础设施建设。落实全省城镇污水、垃圾处理设施建设三年推进方案。完善城镇污水处理系统，完成2个城市生活污水处理厂提标扩容，加快建设城镇污水处理厂（站）和配套管网设施，市区、县城和建制镇污水处理率分别达到94%、80%和48%。实施生活垃圾强制分类，完善垃圾分类设施，加快建设生活垃圾焚烧发电厂等重点项目，推广农村垃圾生态处理“罗江模式”，城市生活垃圾无害化处理率达100%。推进“厕所革命”，新（改）建公厕188座。

坚定走绿色发展之路。大力争创环保模范城市。以“一谷八江十湖”为生态核心，深入实施大规模绿化行动，有序推进县（市）园林城市创建，城市绿化覆盖率达42%。发展绿色循环经济，抓好中德资源循环利用产业园等项目落地落实，建设资源综合利用产业集聚区和国家资源综合利用“双百工程”示范基地。推广绿色建材和装配式建筑，加快建设省级绿色生态示范城区。全面完成国家节能减排财政政策综合示范市任务，建立健全长效机制。

六、着力保障和改善民生，筑牢全面建成小康社会根基

抓住群众最关心最直接最现实的利益问题，既尽力而为，又量力而行，不断满足人民群众日益增长的美好生活需要。

提升民生保障水平。继续实施十大民生工程，精心办好民生实事。打好精准脱贫攻坚战，聚焦“两不愁、三保障”“四个好”目标，加大财政保障力度，下足“绣花”功夫，提高脱贫质量，既不降低标准，也不吊高胃口，激发贫困人口内生动力，确保减贫13469人。深入推进藏区彝区对口帮扶。抓好高校毕业生、就业困难人员、去产能分流职工等重点群体就业，城镇登记失业率控制在4.2%以内。全面建成覆盖城乡居民的多层次社会保障体系，各类社会保险参保率达93%。落实统一的城乡居民基本医疗保险制度，建立常态长效的医保监管制度。推进社会救助，关心关爱留守儿童。加快养老机构提质增效，推动养老服务向村级延伸。实施危旧房棚改7802套，完成农村危房改造5000户。

提高公共服务能力。推进学前教育普惠提质，新建幼儿园11所。推动城乡义务教育一体化发展，深化校际协同共建，新建小学5所，改善农村义务教育薄弱学校基本办学条件28所。促进普通高中特色化多样化发展，推进新高考改革。加快四川城市轨道交通职业学院、四川电影技术职业学院等5所高校的筹建，支持四川天一学院升本。启动名学校、名校长、名教师“三名工程”。加快打造“健康德阳”，实施“名医名院十百千工程”，推广家庭医生签约“1+1+1”模式和罗江“一体化医共体”经验，创建医养结合示范单位1-2家，不断提升卫生计生服务品质。参加省第十三届运动会，新建一批全民健身设施。

加强和创新社会治理。深入推进“平安德阳”建设，着力打造共建共治共享的社会治理新格局。深化“主动警务战略”，加快构建立体化信息化社会治安防控体系。健全预防和化解社会矛盾机制，推动社会治理重心向基层下移，发挥社会组织作用，实现政府治理和社会调节、居民自治良性互动。加强社会信用体系建设，建立健全守信激励和失信惩戒制度。积极防范和化解系统性金融风险、地方政府债务风险。把安全红线作为发展底线，坚决遏制重特大安全事故发生。加强食品药品监管，提升防灾减灾救灾和突发事件应急处置能力，强化国防动员和后备力量建设，确保社会和谐稳定、人民安居乐业。

各位代表！做好今年工作，必须大力加强政府自身建设，推动职能作风新转变、服务效能新提升，建设人民满意政府。一是坚持依法行政。主动接受市人大及其常委会的法律监督、工作监督和市政协的民主监督，认真做好建议提案办理工作。广泛听取民主党派、工商联、无党派人士的意见，支持工会、共青团、妇联等人民团体发挥作用。推进城市管理法律法规贯彻实施。加强重点领域政府立法，依法依规制定规范性文件。深化“七五”普法和法律“七进”。二是优化政务服务。扎实开展“不忘初心、牢记使命”主题教育，坚定理想信念，永葆共产党员为人民服务的本色。深化“互联网+政务服务”，力争让群众办事只跑一趟。推进政务公开、信息公开，畅通信箱、热线、微博等民意诉求渠道。完善政府绩效管理，健全激励和容错机制，从严整治不作为、慢作为、乱作为。三是加强作风建设。深入贯彻落实全面从严治党新要求，巩固拓展落实中央八项规定精神成果。持之以恒正风肃纪，坚持无禁区、全覆盖、零容忍，整治群众身边的腐败问题和作风问题。深化“一诺三清”制度，深入开展走基层“清风行动”，强化行政权力监督，突出发挥“大数据”在监督中的作用，大力营造风清气正、干事创业良好氛围。

各位代表！新时代的嘹亮号角已经吹响，德阳的宏伟蓝图正徐徐展开，时代呼唤担当实干者挺立潮头、奋楫争先。让我们紧密团结在以习近平同志为核心的党中央周围，在省委、省政府和市委的坚强领导下，不忘初心、牢记使命，团结一心、砥砺奋进，坚定走好新时代的长征路，为全面建成高质量小康社会、实现人民对美好生活的向往而努力奋斗！
